# Study of the effect of family communication and function, and satisfaction with body image, on psychological well-being of obese girls: the mediating role of self-esteem and depression

**DOI:** 10.1186/s13034-020-00345-3

**Published:** 2020-10-12

**Authors:** Zabihollah KavehFarsani, Roya Kelishadi, Kioumars Beshlideh

**Affiliations:** 1 Department of Counseling, Shahrekord University, Shahrekord, Iran; 2 Child Growth and Development Research Center, Research Institute for Primordial Prevention of Non-Communicable Disease, Isfahan University of Medical Sciences, Isfahan, Iran; 3 Department of Psychology, Shahid Chamran University of Ahvaz, Ahvaz, Iran

**Keywords:** Obesity, Communication, Body image, Self-concept, Depression, Adolescence

## Abstract

**Background:**

Obesity has become a global problem in childhood and adolescence. The objective of the present study was to investigate the impact of family communication and function, and body image satisfaction, on psychological well-being by considering: the mediating role of self-esteem and depression.

**Methods:**

In this cross sectional study, 173 obese and overweight female students were selected and evaluated based on body image satisfaction, self-esteem, depression, psychological well-being, functioning, and family Communication. The proposed model was evaluated through structural equation modeling, using AMOS and SPSS software.

**Results:**

Results showed that family communication and function directly affected adolescents’ psychological well-being. In addition, family communication and function, as well body image satisfaction indirectly affected psychological well-being through self-esteem and depression.

**Conclusion:**

The current finding suggests that the psychological well-being of obese adolescent girls is associated with many factors, including family functioning and communication, body image satisfaction, self-esteem, and depression. The factors identified in this study may be helpful for mental health policy-makers, in planning and implementing preventive and therapeutic intervention programs.

## Background

Obesity and overweight, especially among children and adolescents, have become a major public health concern in the twenty-first century. The evidence suggests that the prevalence rate of obesity is increasing [1, 2]. In 2016, among children aged 5–19, 50 million girls and 74 million boys were obese, and 213 million children and adolescents were overweight worldwide [3]. Therefore, obesity has become a global problem in childhood and adolescence [4]; obesity in this period is leading to obesity in adulthood [5]. In fact, this group of people is more prone to problems, and negative physical and emotional consequences, including cardiovascular disease, type 2 diabetes, high blood pressure, high cholesterol, sleep disorders, dyslipidemia, depression, social stigmatization, and low self-esteem [6–9].

According to the world health organization adolescence is a period between the ages of 10 and 19 years old, characterized by rapid physical, mental, social and emotional changes [10]. The development of adolescents closely depends on their family [11]. Therefore, some studies have emphasized on the importance of family factors on adolescents’ mental health and well-being [12–14]. However, the investigation of the analyses of obese and overweight children, and adolescents shows that most of the studies are conducted on themes such as physical activity [15, 16], leisure time, parenting style [17, 18], child, mother and family stress, food perception, eating and weight-focused behaviors [19, 20]. However, addressing these issues is not the only approach to help these individuals; therapists should also consider the family and psychological factors of adolescents, especially obese individuals, in their treatment and mental health [21].

Family is the first place, in which the child learns different behaviors and develops expectations for his social life. In this environment, family function and communication play an important role. Family function involves emotional, physical and psychological activities of family members [22], it seems that the poor family function is strongly related to childhood obesity [23, 24]. On the other hand, family communication is characterized by the ways of communication among family members. Poor communication among family members leads to higher levels of obesity in children [25]. Therefore, in the treatment of obesity in children and adolescents, not only it is important to pay attention to the context of families and family ties [21, 26], but also the results of some studies have emphasized on the role of parents and family in the mental health of obese adolescents [27, 28]. In fact, family function and communication can play an important and protective role in reducing depression, increasing self-esteem, and improving the psychological well-being of obese individuals [27, 29]. Kelly et al. observed that in families with better communication, adolescents have high levels of self-esteem [30]. However, obese children are often negatively perceived by their family members; even the research studies on parental feedbacks on their children's obesity showed that many parents responded with anger. This can lead to decreased self-esteem, eating disorder, and increased risk of depression in these adolescents [31, 32].

Another important issue, which is related to the adolescents’ psychological well-being, is body image. Many longitudinal studies showed that those who are not satisfied with their body image are at high risk of depression and low self-esteem [33–35]. In fact, there is a direct inverse relationship between body image dissatisfaction and self-esteem. In other words, if individuals are dissatisfied with their body image, their self-esteem will reduce; this can affect their psychological well-being [36].

Given the gender differences, especially during the pubertal period, we conducted the study only in one gender to reduce the confounding factors. Moreover, as today’s adolescent girls are the future mothers of society; if we want to improve the health of future generations, it is important to pay attention to girls’ physical and mental health. In fact, since weight gain and obesity in girls during adolescence often continue into adolescence, it can also have adverse effects on their pregnancies. Therefore, the authors of this article paid attention to the issue of obesity and overweight in girls during adolescence. The choice of adolescence was also due to the fact that adolescence is a high-risk period for overweight and obesity since it causes fundamental changes in body composition, insulin sensitivity, eating behaviors, activities, and psychological adjustment [37, 38].

it seems that family function and communication, and body image of overweight and obese adolescents, can affect their psychological well-being not only directly, but also through self-esteem and depression. Literature reviews showed that few research studies have examined these variables in a single study, especially through an exploratory model. Therefore, the objective of the present study was to investigate the relationship between family communication and function, and body image, on one hand, and psychological well-being, considering the mediating role of self-esteem and depression, on the other hand. Therefore, the present study investigated the direct effect of family communication and function, and body image, as well as their indirect effect through self-esteem and depression on psychological well-being, in overweight and obese adolescent girls. To do so, the following hypotheses were tested:

1. Family communication and function, and body image have a direct effect on psychological well-being.
2. Family communication and function, and body image have an indirect effect on psychological well-being through self-esteem.
3. Family communication and function, and body image have an indirect effect on psychological well-being through depression.

## Method

The current research was cross-sectional study. Data were gathered using questionnaires; structural equation modeling was used to test the suitability of the proposed model. With regard to the sampling method, from among 10 girls' high schools in Shahrekord, 5 high schools were selected randomly. Then, by referring to those 5 high schools and reviewing the health records of all students along with the health educator of the high schools, the researchers selected individuals who had inclusion and exclusion criteria for this research study. In fact, the inclusion criteria included the participants’ desire to participate in the study, the BMI of each individual ranged between 85 and 95th (i.e., overweight) and more than 95th percentile (i.e., obese individuals), the age range of 12 to 16 years, lack of physical problems and disabilities such as blindness. In addition, the criteria for leaving the study exclusion included not completing the research questionnaires, having underlying diseases such as cardiovascular disease or asthma. Ultimately, the number of individuals selected based on the inclusion criteria was 300. The BMI of students was extracted from their health records. BMI in the health records was calculated by dividing weight (kilograms) by height (square meters). Then, according to the BMI index for age and sex, individuals who were overweight (85th–95th percentile) and those who were obese (> 95th percentile) were selected. BMI percentages for age and sex were based on CDC2000 (CDC: Centers for Disease Control), which was dedicated to the adolescents [39]. Then, the authors selected a sample of at least 20 participants for each parameter following Jackson’s (2003) suggestion for the ideal sample size [40]. This study’s proposed model included ten parameters; a total of 200 individuals were considered as the initial sample of this research study. The researchers randomly selected 200 of 300 girls with obesity and overweight. Then, in order to observe the ethical issues, each individual was explained the objectives of the study. Furthermore, they were ensured concerning the confidentiality of the data, their names and addresses. They were also given the freedom to leave the research study whenever they felt uncomfortable or anxious. If each student agreed to participate in the study, his parents would be informed about the research study, and their consent would be received through telephone. Finally, for the sake of data collection, the questionnaires were distributed among the participants, and they were requested to answer all the questions carefully. It should be noted that the ethical standards of this research study were approved by the ethics committee of Shahrekord University. It should be noted that the questionnaires of this study were distributed among 200 eligible individuals, out of which 173 questionnaires were collected without any problems and all the questions were answered by the subjects.

## Measures

Data were collected, from the Center for Epidemiological Studies, using depression scale for children, Ryff’s scales of psychological well-being, and Rosenberg self-Esteem scale, general functioning scale, body satisfaction and family communication scales.

### Center for Epidemiological Studies Depression Scale for Children (CES-DC)

The CES-DC scale is a short self-report scale designed to measure the current level of depressive symptomatology in the general population. The scale consists of 20 items [41]. Some of these items were modified by Wiseman et al. in order to be used among children and adolescents aged 6 to 17 years. Each item is assigned a score of 0 to 4. A score of 0 was specified for none and 3 for a lot. The score makers considered a cutoff point as above 15 [42]. Barkmann et al. implemented the scale on 2863 German children and adolescents aged 7 to 17 years, and the internal consistency of the scale (Cronbach’s alpha) was reported to be 0.71 [43]. Moreover, in Essau et al. study of 1984 sample of Iranian adolescents aged 12 to 17 years, the results showed a high internal consistency of this scale in the Iranian context (Cronbach’s alpha = 0.87) [44]. In this study, the Cronbach’s alpha coefficient was reported as 0.71.

### Rosenberg Self-Esteem Scale (RSES)

Self-esteem was measured with the Rosenberg self-esteem scale (RSES; Rosenberg 1965). The scale consists of 10 self-report items that express positive feelings of value, or acceptance of themselves. Response options range from strongly disagree (1) to strongly agree (4). In Supple et al. study of 1,248 European-American, Latin, Armenian, and Iranian adolescents with the average age of 15 years, the Cronbach’s alphas for the full scale in these data were 0 0.79, 0 0.82, and 0.86 for the Latin, Armenian/Iranian, and European-American samples, respectively [45]. In Iran, the test–retest correlation coefficients with time intervals of two weeks, five months, and one year were reported to be 0.84, 0.86, and 0.62, respectively [46]. In this study, the Cronbach’s alpha coefficient was reported as 0.86.

### General Functioning Scale

The GFS (Epstein et al. [52]) a subscale of the General Functioning Scale of the McMaster Family Assessment Device, used as a single index for assessing family performance because of its association with other FAD subscales. This subscale consists of 12 items, showing six items of healthy family functioning and six items of unhealthy family function. Each item is given a score of 1 to 4. Bihun et al. stated that this scale can be completed by adolescents over 12 years as well as children in the age range of 12–7. The internal consistency reliability of the GFS is good, with a Chronbach’s alpha of 0.92 [47]. This scale was also used in Shams and Ghanizadeh among children aged 7 to 18 years in the context of Iran [48]. In this study, the Cronbach’s alpha coefficient was reported as 0.66.

### Ryff’s Psychological Wellbeing (PWB) scale

This scale which was designed by Ryff in 1989 has 18 items, scored on a 6-point Likert scale per item. It is a scale of six components (self-acceptance, environmental mastery, positive relationship with others, and purpose in life, personal growth, and autonomy) that measures overall psychological well-being. The six-dimensional psychological characteristics reported by Ryff, varied from 0.86 to 0.93 [49]. In Lavasani’s, et al. study of 398 female high school students in Iran, the Cronbach alpha coefficients of 6 components of this scale (i.e., self-acceptance, environmental mastery, positive relationship with others, and purpose in life, personal growth, and autonomy) were reported as 0.79, 0.71, 0.70, 0.54, 0.61, 0.72, respectively [50]. In this study, the Cronbach’s alpha coefficient was reported as 0.75.

### Body satisfaction

This scale consists of two items, which measure the extent to which adolescents are satisfied with their weight and appearance. The question was ‘‘Please tell us how much you agree with these: (a) I am happy with my weight; (b) I am happy with how I look”. This is a 5-point Likert scale, ranging from strongly disagree (1) to strongly agree (5). This scale was used in one study [51]; the estimated Cronbach’s alpha coefficient at two time points were between 0.67 and 0.71 [51]. In this study, the Cronbach’s alpha coefficient was reported as 0.65.

### Family communication scale

This scale, which is one of the McMaster Family Assessment Device dimensions, consists of six items. Communication, which is defined as the exchange of information among members. It is a 4-point Likert scale, ranging from strongly disagree (1) to strongly agree (4). Reverse scoring is used for some items. The scores of the items are added. A higher score indicates a better and more positive family communication. In the study Epstein et al. the Cronbach’s alpha coefficients were reported 0.75 [52]. The original scale was in English; the subscale of communication of this scale was carefully translated to Persian by the researcher. It was given to two individuals who were fluent in both Persian and English to translate it back to English using reverse translation method. Then, they were asked to re-translate it to Persian. The content validity of this subscale was examined by specialists (i.e., psychologists and family counselors). A number of limited modifications were made. Finally, after several stages of investigation, review, modification and implementation, the Persian form of this subscale was prepared for the research study. It was piloted on several girls, and finally it was ready to be used in the present study. In this study, the Cronbach’s alpha coefficient was reported as 0.72.

### Statistical analysis

The present research data were analyzed through statistical indicators and methods such as mean, standard deviation, and Pearson correlation coefficient; in order to investigate the proposed research model, structural equation modeling was used, and to test the indirect effects of the variables.

Structural equation modeling was used to test the proposed model of the relationship between family communication and function, and body image, on one hand, and psychological well-being mediated by self-esteem and depression, on the other hand. Structural equation modeling is a form of causal modeling that includes a diverse set of mathematical models, computer algorithms, and statistical methods that fit networks constructs to data [53]. The AMOS version 23 was used and the interpretation of the bootstrap data was accomplished by determining whether the 95% bias-corrected and accelerated confidence intervals (BCa 95% CI) did not contain zero. The analyses and bootstrap estimates were based on 5000 bootstrap samples.

## Results

### Demographic findings

About 46.8% of the participants were in the age range of 12 to 14 years, and 53.2% were between 15 and 16 years. Their parents' educational level ranged from illiterate to postgraduate. Approximately, 9.2% of the participants reported their economic level as very poor, 39.9% moderate, 39.9% good, and 11% reported as excellent. Moreover, 3.5% of the student’s fathers were unemployed, 38.7% employed and 57.8% were entrepreneur.

Descriptive statistics (i.e., mean, standard deviation and correlation between the variables) are shown in Table 1. As shown, except for the relationship between family communication and body image, and that of body image and psychological well-being, the variables were significantly correlated at the 0.001 and 0.05 significance levels. Therefore, regarding the first hypothesis, it should be stated that, except the relationship between body image and psychological well-being, in which p was less than 0.206, other relationships were significant.

**Table 1:** The descriptive statistics for independent, mediator and dependent variables among obese and overweight girls

Variables	Descriptive statistics							
	Mean	Std. deviation	Correlations among variables					
			1	2	3	4	5	6
1. Body image	6.26	3.45	–					
2. Depression	42.79	10.16	− 0.26**	–				
3. Self- esteem	30.24	6.39	0.24**	− 0.53**	–			
4. Family communication	17.63	4.74	0.77	− 0.35**	0.43**	–		
5. Family function	35.20	5.94	0.15^*^	− 0.47**	0.53**	0.44**	–	
6. Psychological well-being	77.06	12.51	0.09	− 0.53**	0.60**	0.47**	0.54**	–

^*^ p < 0.05; ** p < 0.001

The proposed model in this study included six variables, three of which were predictor (independent) variables, one was the dependent variable and two were mediating variables. Proposed model fit was based on the combination of fit measures (see Table 2). The ratio of χ^2^ to degree of freedom is 1.535); the acceptable range is between 2 and 3. The value less than 2 is considered very appropriate. Another important index is RMSEA, which was 0.05 in this study. Since the acceptable range is between 0.05 and 0.07, this study’s index is acceptable. Other model-data fit indices in this study showed the overall fit of the model to the available data (CFI = 0.967, NFI = 0.914, IFI = 0.968, TLI = 0.948).

**Table 2:** Proposed model fit based on fit indices

*χ*^2^	df	(*χ*^2^/df)	CFI	IFI	NFI	TLI	RMSEA
53.723	35	1.535	0.967	0.968	0.914	0.948	0.05

**Fig. 1:**
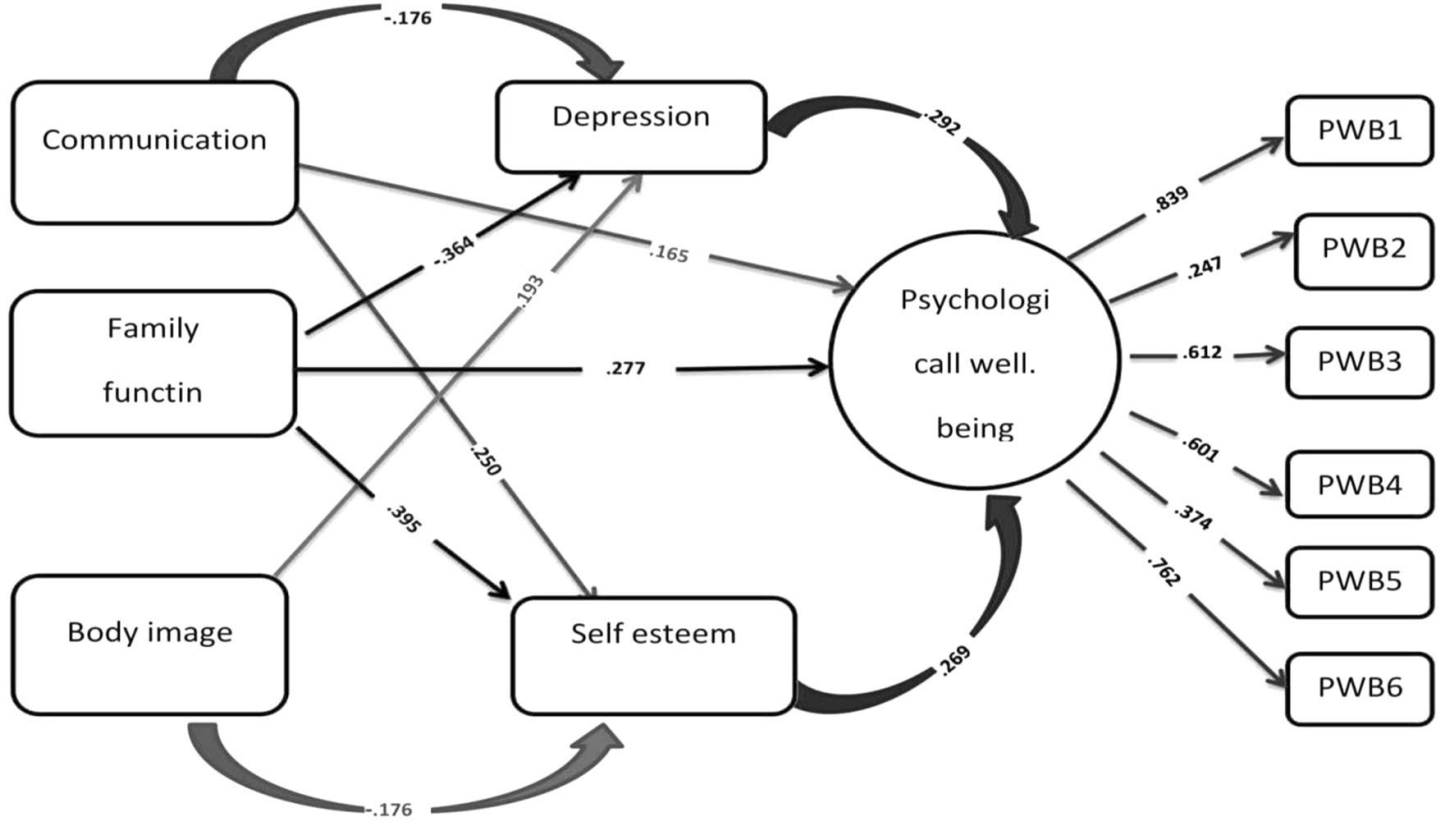
The proposed model of the present study along with the standard path coefficients

As shown in Fig. 1, the path coefficients of communication to psychological well-being (P = 0.012, β = 0.165) and family function to psychological well-being (P = 0.001, β = 0.277) are statistically significant; i.e. the path coefficients of these two variables are significant. However, path coefficients for body image to psychological well-being (P = 0.127, β = − 0.092) are not statistically significant. Therefore, in regards to the first research hypothesis, other than the path of body image to psychological wellbeing, the first hypothesis is confirmed with confidence of 95%.

Regarding the second and the third hypotheses, the results of Bootstrap for the mediated paths of the proposed model can be seen in Table 3. As shown in Table 3, the second and the third hypotheses of this study are supported. In other words, all pathways of family communication and function and body image to the psychological well-being through the mediating variables of self-esteem and depression, were statistically significant. Thus, depression and self-esteem acted as mediating variables between independent and dependent variables. It should be noted that for analyzing this part, each dependent variable, a mediator and an independent variable were examined separately.

**Table 3:** Data regarding effects

	Total effects	Direct effects	Indirect effects	Lower bounds	Upper bounds	P value
Family function → Self-esteem → Psychological well-being	1.146	0.657	0.489	0.340	0.662	0.000
Family function → depression Psychological well-being→	1. 146	0.796	0.350	0.231	0.511	0.000
Communication → depression Psychological well-being→	1.262	0.878	0.384	0.244	0.577	0.000
communication → Self-esteem → Psychological well-being	1.262	0.700	0.562	0.385	0.771	0.000
Body image → Self-esteem → Psychological well-being	0.350	− 0.197	0.547	0.270	0.840	0.000
Body image → depression Psychological well-being	0.350	0.167	0.516	0.291	0.820	0.000

## Discussion

The present study examined the relationship between family communication and function, and body image, on one hand, and psychological well-being mediated by depression and self-esteem, on the other hand, among overweight and obese girls. The results showed that family communication and function directly and indirectly (i.e., with the mediation of depression and self-esteem) were correlated with psychological well-being. In addition, although the body image was not directly correlated with psychological well-being, it was correlated with psychological well-being through the mediating variables of depression and self-esteem. The findings of the study suggest that to improve the psychological well-being of obese and overweight adolescents, family issues and satisfaction with body image should be taken into account. Although the findings of this study do not indicate causality, they can be used in assisting the psychological well-being of this particular group.

During adolescence, and especially from the age of 13, girls are twice as likely as boys to develop depressive symptoms, and this difference continues into adulthood [54]. Another study found that adolescent girls with obesity and overweight have more depressive symptoms than normal-weight girls [55]. The study of Mannan et.al. indicated that depression in adolescents can increase the risk of obesity by 70%, while obesity in adolescents might increase the risk of depression by 40%. Moreover, their findings indicated that among girls at the start of adolescence, the risk of depression resulting in obesity is significantly higher than that of the risk of obesity resulting in depression. Therefore, according to their findings, a two-way relation exists between depression and obesity, where various factors such as biology, lifestyle, as well as behavioral and environmental factors might cause this two-way link [56]. In this context, it can be stated that since adolescent girls are more sensitive to interpersonal relationships, including relationships with their families, family stress and conflict can be a very strong predictor of depression in girls [55]. That is why depression is referred to as an interpersonal disease; the role of family and the related factors as the predictor of depression are especially prominent in adolescent girls [57]. Thus, we considered depression as a mediator variable. Various studies have shown that families with characteristics, such as low cohesion levels [58], high conflicts between children and parents [59, 60], and high levels of anxiety and control [61], increase the probability of depression in their teens; these families have poor family functioning; this can lead to a lot of stress in the family environment, which in turn affects the adolescents’ depression [62]. On the other hand, ineffective parent-adolescent communication has been identified as a factor which increases the risk of depressive symptoms in adolescents [63]. Therefore, since the greatest conflict between parents and children occurs during adolescence [64], if the family does not have good performance and communication and can not manage the stressors and conflicts well, there will be the increased risk of depression in adolescents, especially adolescent girls, which is due to sensitivity to their interpersonal relationships; this depression also jeopardizes their psychological well-being. In this vein, the results of Sheck’s study on Chinese adolescents showed that there is a significant relationship between family performance and psychological well-being [65]. Moreover, the findings of the present study are in line with those of Sheeber et al. and Sze, et al. Sheeber et al. who reported that when there are more negative communication patterns between parents and adolescents in the family, and family members do not support each other sufficiently, there is a high risk of depression [66]. Sze et al. study found that close families with greater and higher coherence, communication, and support have lower levels of depressive symptoms in their adolescents [67].

Self-esteem is another variable which had a mediating role between communication and family functioning and psychological well-being in this study. Childhood period places an important role in shaping the children and adolescents’ self-esteem. Bowlby, in the Attachment Theory, emphasized the importance of mental health and psychological well-being for attachments security [68]. In other words, the main caregivers is available, supportive and sensitive in times of need, it can result in forming of secure attachments in children [69]. According to the attachment theory, secure individuals have higher self-esteem compared to insecure ones since they are quicker to experience social interactions. Since in secure attachments, caregivers always provide suitable feedbacks at appropriate times, children to develop feelings of trust and dependence to their caregivers while also forming a positive self-concept through constant and predictable feedbacks from their caregivers; seeing themselves as a likable individual, which in turn results in higher self-esteem [70]. The self-esteem formed during childhood can continue throughout the adolescence. Thus, family is one of the most important factors influencing adolescents' self-esteem; it can influence adolescents' self-esteem through the relationship between parents and children, parenting styles and even siblings' relationship with each other [71]. Therefore, the role of communication and family performance on adolescents’ self-esteem, especially among of girls, is important and fundamental; on the one hand, Bolognini et al. study showed that girls' self-esteem is globally lower than that of boys [72]. On the other hand, another study showed that when adolescents perceive many conflicts within the family, their self-esteem would decrease and their depression would increase [73]. Furthermore, the result of Preechawong et al. research study showed that family function is a strong predictor of adolescents’ self-esteem [74]. Therefore, to increase self-esteem, decision-makers should improve family communication and function by giving lectures, having a friendly relationship with adolescent children, expressing affection, listening to problems and concerns, solving existing conflicts and striving to make the family environment more relaxing.

Another independent variable in this study was body image. The findings showed that body image have a negative relationship with depression, and a positive relationship with self-esteem. It is also related to psychological well-being through the two mediating variables.

Body image development occurs in communities, where a set of standards for attractiveness, body weight and body shape are defined. These standards are ideally transferred to the audiences through the media, which is responsible for conveying the message. Consequently, for adolescents who are in the midst of rapid physical, mental and social changes, there is a conflict between these standards transmitted from culture and the media and their body reality; research studies have shown that this has a negative effect on body image acceptance and body satisfaction, especially in adolescent girls [75, 76], This increases the risk of decreased self-esteem and increased depression among these individuals [33]. The results of this study showed that the more satisfied overweight and obese adolescent girls are with their body image, the less depressed they are, and as a result, they have high self-esteem. However, the results of Faubel’s study showed that there is a population of obese women who are satisfied with their bodies; their psychological behavior is not significantly different, compared to other women [77].

It should be noted that what distinguishes the results of this study from those of previous research studies is the fact that in this study all these variables were used simultaneously in a structural equation model. Direct and indirect relationships of the predictive variables on the criterion variable, which is psychological well-being, were also shown. In addition, this study examined the role of family variables (e.g., family functioning and communication in adolescence) in psychological well-being both directly and indirectly (through the variables of depression and self-esteem). In previous studies, these items were not thoroughly studied.

## Conclusion

Our data provide an exploratory model, showing that mental health authorities, schools and parents should consider the role of mediating and independent variables of this study, in order to improve the psychological well-being of adolescent girls, especially the obese and overweight. There must be proper communication between parents and children in the family context; family members should support each other and solve their conflicts through conversation. In other words, it is advised that parents have friendly relationships with their children. Furthermore, with regard to the sensitive period, in which adolescents are exposed to, it is recommended to help them accept their body image.

## Limitations and strengths of the study

The main limitation of the study is the cross-sectional design, which lacks a control group. Another limitation of this study is the use of structural equation modeling, which does not prove causality. Finally, the instruments used in this study were self-report questionnaires with the associated specific limitations. However, one of the strengths of this study is that it is the first study conducted in Iran in this context, which put the abovementioned variables into a structural equation modeling. In addition, the study emphasizes on the role of family variables to improve psychological well-being, which is usually ignored.

This research study was conducted in the context of Iranian population. However, as the review of the literature showed, there were some similarities between the results of this study and those of the previous ones. These similarities include the role of family variables and body image satisfaction. In this study, similar to other studies, it was confirmed that family variables during adolescence play a very important role in increasing self-esteem and reducing depression. Moreover, Iranian teenage girls’ satisfaction from body image was similar to other teenagers in other countries. Body image is an important variable for adolescent girls, and it is associated with self-esteem and depression. Therefore, the researchers provided suggestions for the future studies. This study can be conducted on boys with obesity and overweight as well as those with normal weight. In fact, performing structural equation models is an introduction to clinical and experimental works.

## Data Availability

All data and material are available at the Department of Counseling, Shahrekord University.
